# Utilizing transformative learning theory to enhance professional identity formation

**DOI:** 10.3389/jpps.2025.14605

**Published:** 2025-06-05

**Authors:** Christine Pan, Jennie B. Jarrett, Kathryn Sawyer

**Affiliations:** Department of Pharmacy Practice, Retzky College of Pharmacy, University of Illinois Chicago, Chicago, IL, United States

**Keywords:** transformative learning theory, professional identity formation, pharmacy education, self-reflection, case vignette

## Abstract

**Objective:**

To analyze the impact of a Transformative Learning Theory (TLT)-based toolkit on pharmacy students’ self-evaluation of professional identity formation (PIF).

**Methods:**

This prospective, interventional cohort study included pre-clinical pharmacy students in a hospital skills-based course. Study participants were included if they completed the Professional Self Identity Questionnaire (PSIQ-9) and Macleod Clark Professional Identity Scale (MCPIS-9) at baseline (week 1), midpoint (week 8), and endpoint (week 15) of the course. The primary outcome was to assess the mean change in PSIQ-9 and MCPIS-9 scores from baseline to endpoint; the outcome was analyzed using the Wilcoxon-Signed Rank Test. Secondary outcomes included assessing the mean difference in questionnaire scores from baseline to midpoint and midpoint to endpoint.

**Results:**

Seventy-nine pharmacy students were eligible, with 11 (14%) completing both questionnaires at all time points and 39 (49%) completing them at baseline and midpoint. Comparing baseline and endpoint scores, there was an increase in the PSIQ-9 mean difference for teaching others and a decrease in the MCPIS-9 for feeling ashamed of the profession. No MCPIS-9 differences were found between baseline and midpoint. Three PSIQ-9 questions, communication, using patient records, and teaching others, were significant at baseline and midpoint.

**Conclusion:**

The TLT-based toolkit had a minimal impact on students’ self-evaluation of PIF based on the PSIQ-9 and MCPIS-9 questionnaires over a 15-week course. Studies with larger sample sizes and longer durations are needed to provide more conclusive results.

## Introduction

Professional identity formation (PIF) is the active process of integrating the knowledge, skills, values, and behaviors necessary to be a proficient professional [1]. In 2014, a PIF task force was established by the American Association of Colleges of Pharmacy (AACP) Council of Deans, which released a report defining PIF as a transformative process of discovering and adopting the behaviors and interactions associated with a professional role, leading to personal growth and integration [1]. The transformative nature of PIF has been well described in medical education literature, notably by Jarvis-Selinger and colleagues, who characterize PIF as a developmental process involving shifts in meaning-making and self-concept that occur through experiences and socialization into the profession [2]. These shifts are not automatic; they require intentional reflection and guided experiences. As such, students’ PI is expected to evolve throughout their professional training, shaped by both curricular and co-curricular experiences.

One of the challenges to successful PIF is the need for students to reconcile their ideal professional aspirations with the realities of practice [3]. An underdeveloped PI may contribute to post-graduation challenges, including difficulty navigating uncertainty and evolving responsibilities, which in turn may increase vulnerability to dissatisfaction or burnout [4]. Therefore, an explicit approach to supporting PIF across the pharmacy curriculum is essential to ensure graduating students think, act, and feel like pharmacists [1].

There is no universally accepted best-practice model for developing PIF in pharmacy education [1]. Previous research into pharmacy curricula approaches to improve PIF includes reflection, coaching, socialization activities, mentorship, discussion, and guided practice. These approaches have been grounded in six learning theories: self-authorship, provisional selves, self-determination, social identity, social learning, and being-in-the-world [5–15]. Another grounded approach, currently missing from pharmacy education literature, is Mezirow’s Transformative Learning Theory (TLT) [16]. TLT is an andragogical approach developed for use with adult learners to build meaning through self-reflection. The theory focuses on the critical reflection of competing beliefs, behaviors, and values. The foundation of TLT is to expand the learner’s consciousness through a disorienting dilemma that challenges their feelings and assumptions to build new perspectives and purpose [16]. Since PIF is an active process of an individual internalizing what it means to be a professional, structured reflective exercises using TLT as the grounded theory could potentially enhance PIF.

## Literature review

### Professional identity formation

Currently, PIF among pharmacy students has been studied worldwide using questionnaires [7–9], interviews [10, 11], focus groups [12, 13], reflective essays [14, 15] and personal narratives [17]. These studies concluded that pharmacy educators need to consider how to address the variation in how pharmacy students understand professional practice [8] and identify variables that could lead to the increased development of PI, such as having pharmacy experience before starting pharmacy school [7]. Interventions like work-integrated learning with coaching [9] and using innovative strategies such as “role-emerging placements” [10] can improve students’ PIF. More innovative methods of studying PIF in pharmacy students are needed to assess their PIF development over time.

In a study conducted by Bernabeo et al., a set of vignettes involving challenging professional situations was developed, and 40 practicing physicians with relatively diverse experiences completed these vignettes [18]. The study found that the vignettes prompted reflection on the intended areas and a reflective approach allowed physicians to engage with the complexities of their work. This suggests that similarly structured, challenging case vignettes could be an effective tool to foster PIF among pharmacy students by encouraging critical reflection on real-world dilemmas.

### Mezirow’s transformative learning theory

Currently, multiple student development theories have been used to inform interventions geared toward PIF, such as self-determination theory [6], social identity theory [15], Baxter-Magolda’s self-authorship theory [14], Crenshaw’s theory of intersectionality [19] and more. One theory missing from pharmacy literature is the use of Mezirow’s TLT [16]. The use of TLT has been studied within medical education with positive results. To date, TLT has been utilized as an andragogical approach in practice-based quality improvement curricular design within medical education. A scoping review performed by Vipler et al. analyzed 28 articles that mentioned “transformative learning” or “transformational learning” and involved those in graduate medical education [20]. None of the articles included trainees from the pharmacy profession. One of the largest themes found in this scoping review was the strong relationship between transformative learning and professionalism. Given that reflection is a foundational component of transformative learning, this connection highlights the potential for structured reflective activities to serve as a catalyst for professional identity formation in pharmacy students. See Appendix A for the ten phases of Mezirow’s TLT in the process of simulation-based learning in healthcare education.

### Professional identity questionnaires

The Professional Self Identity Questionnaire (PSIQ-9) and the Macleod Clark Professional Identity Scale (MCPIS-9) are validated tools that have been used to assess self-perceived PI among health professional students. Originally developed and validated in cohorts of health and social care students in the United Kingdom, the PSIQ-9 was designed to explore how different educational experiences shape identity formation, while the MCPIS-9 aimed to measure variability in PI among students from various disciplines [21, 22]. Each instrument consists of nine items scored on a 5-point Likert scale.

While these tools have been applied in early studies of PI, particularly in medicine and nursing, their use has become more limited in recent years. In addition, the use of quantitative tools to measure the complex and deeply personal process of PIF has been critiqued. Matthews et al. emphasized the need for caution in interpreting quantitative results related to PIF, noting that such tools may not fully capture the nuanced, reflective, and contextual nature of identity development [23]. Similarly, Garza et al. argue that while quantitative instruments can offer a snapshot of students’ self-perceptions, they may be insufficient when used in isolation to assess the impact of PIF interventions, particularly those grounded in transformative learning [24].

In selecting the PSIQ-9 and MCPIS-9 for this study, the goal was to employ accessible, previously validated instruments that would allow for comparison to existing literature and provide a standardized metric for gauging self-perceived changes in PI. However, it is acknowledged that these tools may have limitations in detecting change following a brief, curriculum-based intervention grounded in Mezirow’s TLT. The PSIQ-9 and MCPIS-9 focus primarily on general perceptions of PI and do not include items that assess critical reflection, meaning-making, or transformative shifts in perspective. As such, while these instruments offered a practical means of capturing baseline and post-intervention perspectives, they may not have been optimally aligned with the reflective and experiential dimensions of the disorienting dilemma-based learning strategy used.

## Methods

### Study design and procedure

This single-center, prospective, interventional-cohort study included second-year pharmacy (P2) students enrolled in the Fall 2023 semester of an introductory to pharmacy practice (IPPE) hospital skills-based course at a large-public-urban college of pharmacy. Seventy-nine students were eligible to participate based on course enrollment. The concepts of TLT and PIF were introduced via a didactic lecture and activity on the first day of class. After the lecture, the research team presented the study protocol and requested student participation. Participation was elicited at three points: week 1 (baseline), week 8 (midpoint), and week 15 (endpoint) of the course. Class time was provided for each data collection point, and an email reminder was sent 1 week after each class period. Refer to Table 1 for the questions and answer choices included in both questionnaires.

**TABLE 1:** Professional identity formation assessment tools.

Professional Self Identity Questionnaire (PSIQ-9) Questions	PSIQ-9 answer choices
P1. When I am working with other health and social care professionals, I feel like a:	0 = N/A 1 = First-day student pharmacist 2 = First day of P3 year 3 = First day of P4 year 4 = Last day of P4 year 5 = Newly licensed pharmacist
P2. When I am communicating with patients, I feel like a:	
P3. When assessing a patient, I feel like a:	
P4. When engaging with others in culturally diverse health care environment, I feel like a:	
P5. When I am considering ethical or moral issues, I feel like a:	
P6. When consulting/using patient records, I feel like a:	
P7. When I find myself in an emergency involving a patient, I feel like a:	
P8. When reflecting on my practice (experiences) to identify my learning needs, I feel like a:	
P9. When teaching others, I feel like a:	

Macleod Clark Professional Identity Scale (MCPIS-9) Questions	MCPIS-9 Answer Choices
M1. I feel like I am a member of the pharmacy profession	1 = Strongly disagree 2 = Somewhat disagree 3 = Neither agree nor disagree 4 = Somewhat agree 5 = Strongly agree
M2. I feel I have strong ties with members of the pharmacy profession	
M3. I am often ashamed to admit that I am studying for the pharmacy profession^a^	
M4. find myself making excuses for belonging to the pharmacy profession^a^	
M5. I try to hide that I am studying to be part of the pharmacy profession^a^	
M6. I am pleased to belong to the pharmacy profession	
M7. I can identify positively with members of the pharmacy profession	
M8. Being a member of the pharmacy profession is important to me	
M9. I feel I share characteristics with other members of the pharmacy profession	

^a^ Negatively phrased.

Students enrolled in the course were required to complete the TLT-based PIF toolkit. Students were asked to read an assigned disorienting dilemma case vignette and respond using the TLT-based reflective questions (Table 2). These reflections were conducted via an asynchronous video recording platform in groups of four or five. Students recorded their own responses, listened to their peers’ responses, and then reflected again after hearing their peers’ perspectives. The three-week reflective process occurred four times, a total of four vignettes assigned, during the 15 weeks of the semester. Each week, the students independently completed the reflective process—spending approximately 10 min per week on the activity. The research questionnaires were administered at baseline before any interaction with the toolkit, at the midpoint after completing two vignettes, and at the endpoint after completing four vignettes.

**TABLE 2:** TLT-based toolkit.

Disorienting dilemma case vignettes	
Patient Care Provider Domain	
Improving Medication Adherence	You are working in an outpatient clinic and have an appointment with Mr. S, a 42-year-old Black male patient with a past medical history of intellectual disability, HTN, depression, ESRD on dialysis, and HIV. Mr. S reveals that he was incarcerated for 15 years due to child molestation allegations, during which he contracted HIV. He harbors strong feelings of resentment and anger about his HIV status. Referred to your clinic for resistance to multiple HIV regimens, owing to poor medication adherence; your role is to collaborate with him in improving adherence. Despite your efforts to organize his medications into a monthly pillbox during appointments, Mr. S frequently returns the box at the end of the month with over 50% of the medications untouched. His rationale for non-adherence is simply, “I just don’t want to.”
Helping While Learning	During a 4-week gastroenterology ambulatory care rotation as a new pharmacy resident, you’ve been closely monitoring a patient’s transition to a new Ulcerative Colitis medication. Over this period, he’s been experiencing frequent bloody stools. You’ve continually assessed the situation and advised him to seek emergency care if his condition worsens. Collaborating with your pharmacist preceptor and MD, you’ve adjusted his steroid regimen. Upon starting the new medication, he reported positive results. However, on a Saturday morning while on staff duty, you received an in-basket message directly from the patient. He informed you of recurring frequent bloody stools, despite being on a steroid taper and continuing the new medication. He reached out only to you, not your preceptor or the MD.
Interprofessional Team Member Domain	
Diverse Team Perspectives	As a clinical pharmacist in the trauma ICU, you’re an integral part of a diverse multidisciplinary team overseeing patient care. The team comprises a pharmacist (yourself), an attending trauma surgeon, a third-year surgery resident, a first-year medical resident, a dietician, a respiratory therapist, a social worker, a physical therapist, and a bedside nurse. A patient on the unit is currently facing substantial pain. During rounds, most team members agree on the best course of action, but your perspective on enhancing pain management differs from that of your colleagues
Responsible but No Authority	You are working in an ambulatory care clinic on a collaborative interprofessional healthcare team. You are responsible for seeing patients for chronic disease state management and instructing residents and students on rotation. A physician colleague consults you regarding a patient. After evaluating and discussing the case, you provide a recommendation, but the physician chooses to prescribe a newer medication they learned about from a recent commercial. The physician initiates the patient on the alternative medication and sends a referral for you to follow-up with the patient. You are tasked with managing this patient’s chronic condition in coordination with the team
Information Master Domain	
Patient Advocacy Amidst Tension	As a new hospital pharmacist, you oversee antibiotic stewardship and renal dosing according to hospital protocols. Your responsibilities include assessing patients on antibiotics during shifts, adjusting dosages based on renal function, and suggesting antibiotic de-escalation following sensitivity results. You recently modified antibiotic doses for a patient due to declining renal function in line with approved protocols. You messaged the attending infectious disease physician about the adjustments as a professional courtesy. You come back to your shift next day to find a reply from the physician stating, “I don’t want pharmacists looking at my patients or making recommendations or changes to any of the medications I manage.”
Practice Manager Domain	
Managing a Challenging Team Member	As a new hospital pharmacist, you oversee operations during your shift alongside a consistent team of three technicians. While two technicians excel and collaborate effectively, one technician consistently arrives late, makes errors, and disputes your decisions. Your role entails overseeing all aspects of the shift, making you accountable for the team’s performance and outcomes
Self-Developer Domain	
When Things Don’t Go as Planned	You have always performed super well academically and professionally. You are near the top of your class in pharmacy school, serve on multiple student committees, and work as a pharmacy intern at a local hospital. All your hard work seems to have paid off, since you were invited to interview at all ten of the residency programs you applied to. You feel that all your interviews went really well, and you are excited to learn where you matched. It is finally match day; you are nervous but cannot wait to find out where you will go next year. You open your email; you didn’t match
What Now?	You’ve consistently excelled in pharmacy school, maintaining top performance, and helping others with exam preparation. Balancing studying, research, leadership roles, and social activities has been a strength for you. After graduation, you dedicate free time to studying and pass the NAPLEX and two MPJEs on the first try, providing a sense of relief. However, upon matching to a residency in Illinois, you face the need to take another MPJE. Amid rotations, you diligently study, create flashcards, and review materials from friends and mentors. Two weeks after the exam, a letter arrives with the disheartening word “FAIL.”
TLT-based Reflection Questions	
Question 1	What was your initial response to this professional identity vignette? What emotions or thoughts did it evoke?
Question 2	Explore the underlying beliefs, values, and assumptions influencing your initial response to the professional identity vignette. How do these beliefs shape your perspective?
Question 3	Analyze the professional identity vignette provided. How does it resonate with your own experiences? Which aspects of the vignette challenge your current understanding or viewpoints of what it means to be a pharmacist?
Question 4	Consider the professional identity vignette from another perspective. What perspective did you consider? What areas of tension and common ground can you identify between this alternative perspective and your initial response?
Question 5	Consider your initial response and the alternative perspective. How does this alternative perspective affect your thoughts on what it means to be a pharmacist? What knowledge, skills or ways of thinking may you need to acquire to strengthen your professional pharmacist identity?
Question 6	Watch a peer’s reflective video. What similarities and differences did you see between your initial response and your peer’s?
Question 7	Consider your background beliefs, values, and assumptions versus your peers. How did these beliefs, values and assumptions differ? Can you identify any common themes and variations?
Question 8	Analyze the initial and alternative perspective your peer presented versus your own. How did these perspectives challenge your analysis of the professional identity vignette? How did their analysis affect your thoughts on what it means to be a pharmacist?
Question 9	Watch all peer responses to your initial video. Reflect on how the insights gained from this discussion can be applied in your personal, academic, or professional life. What personal growth and expanded understanding resulted from this process? How did the discussion change how you see yourself as a future pharmacist? How might you navigate future professional identity/disorienting dilemmas more effectively?

### TLT-based PIF toolkit

The toolkit was crafted through an iterative process by the principal investigator. Initial focus sessions, with practicing pharmacists, led to the creation of eight disorienting dilemma vignettes derived from the AACP Core Entrustable Professional Activities (see Table 2). The TLT-based PIF tool kit was then evaluated by PIF experts, individuals who have written or spoken on the topic of PIF within the pharmacy educators’ academy, for alignment with pharmacist PI values and beliefs.

### Participants

Inclusion criteria were pharmacy students enrolled in the course who were over the age of 18 years, able to consent to research, and had completed the TLT-based PIF toolkit as well as the PSIQ-9 and MCPIS-9 questionnaires at baseline and midpoint, or at baseline, midpoint, and endpoint. There were no exclusion criteria beyond those who did not meet the inclusion criteria; however, students who had incomplete questionnaire were not included in the results.

### Outcomes

The primary outcome was to assess the change in PI as measured by the mean difference in scores of student pharmacists’ questionnaire responses from baseline to endpoint regarding their PI after completing the TLT-based PIF toolkit. The secondary outcome was to assess the growth of PI over time as measured by the mean difference in scores from baseline to midpoint and from midpoint to endpoint.

### Statistical analysis

Basic descriptive statistics were used to analyze students’ baseline characteristics. The Wilcoxon signed-rank test was employed to evaluate the nonparametric-paired, ordinal data collected from the PSIQ-9 and MCPIS-9 for both the primary and secondary outcomes. Additionally, descriptive statistics were used to analyze the responses from the PSIQ-9 and MCPIS-9. To examine individual-level trends not evident in group-level analyses, we descriptively coded item-level score changes as development (any increase in Likert score from baseline to endpoint), stasis (no change in score), or regression (any decrease in score), following an adaptation of King et al. [25]. For example, a change from 3 to 4 was coded as development, while a change from 5 to 2 was coded as regression.

## Results

At the baseline, midpoint and endpoint, 49, 75, and 25 students completed both the PSIQ-9 and MCPIS-9 questionnaires, respectively. A total of 11 students completed both questionnaires at all three time points. Due to discrepancies in self-reported demographic information between baseline, midpoint, and endpoint, only the characteristics from the baseline were described. Among these 11 students, the majority were female (72.7%), aged 22–27 years (90.9%), Caucasian (45.5%), held a Bachelor’s degree (81.8%), and had prior pharmacy experience before entering pharmacy school (81.8%). See Table 3 for the full details of the baseline demographics. An additional 28 students completed both questionnaires at only the baseline and midpoint. Their baseline characteristics were similar to those of the 11 students who completed questionnaires at all three time points.

**TABLE 3:** Baseline demographics (n = 11).

Baseline characteristic	n (%)
Gender	
Female	8 (72.7)
Male	3 (27.3)
Age	
Less than or equal to 21 years old	1 (9.1)
22–24 years old	5 (45.5)
25–27 years old	5 (45.5)
Ethnicity/Race	
African-American	0 (0)
Asian	3 (27.3)
Caucasian	5 (45.5)
Latino or Hispanic	1 (9.1)
Two or More	2 (18.2)
Highest degree or level of education completed	
Pre-pharmacy requisites	2 (18.2)
Bachelor Degree	9 (81.8)
Having pharmacy experience before entering pharmacy school	
Yes	9 (81.8)
No	2 (18.2)
Number of years of pharmacy experience before entering pharmacy school	
<1 year	1 (9.1)
1 year	4 (36.4)
1–2 years	4 (36.4)
N/A	2 (18.2)
Currently working in a pharmacy	
Yes	9 (81.8)
No	2 (18.2)
Number of hours of pharmacy work per week	
<10 h/week	6 (54.5)
10–20 h/week	2 (18.2)
N/A	3 (27.3)

### Primary outcomes

Among the 11 student pharmacists who completed both questionnaires at baseline, midpoint, and endpoint, most of their PSIQ-9 responses did not change significantly from baseline to endpoint after completing the TLT-based PIF toolkit; only one question showed a difference. The mean increase in scores from baseline to endpoint ranged from 0 to 0.64 across all nine components of the PSIQ-9, with a difference showing PI growth found in the question related to teaching others (+0.64 [p <0.05]). No other questions showed significant differences in responses. Most students demonstrated PIF development on questions about communicating with patients, considering ethical or moral issues, and teaching others. Most students exhibited PIF stasis when asked about working with other healthcare professionals, using patient records, and being involved in a patient emergency. Students showed equal amounts of PIF development and stasis regarding questions related to assessing a patient, engaging with others in a culturally diverse healthcare environment, and reflecting on experiences to identify learning needs. See Table 4 for all primary outcome results.

**TABLE 4:** Primary outcome from baseline to endpoint (n = 11).

	Mean change (±SE)	*P* Value	No. (%) of students development	No. (%) of students stasis	No. (%) of students regression
PSIQ-9					
P1.	0.27 (0.79)	0.35	3 (27.3)	7 (63.6)	1 (9.1)
P2.	0.36 (1.29)	0.25	6 (54.5)	4 (36.4)	1 (9.1)
P3.	0.18 (1.17)	0.37	5 (45.5)	5 (45.5)	1 (9.1)
P4.	0.55 (0.93)	0.11	5 (45.5)	5 (45.5)	1 (9.1)
P5.	0.18 (1.17)	0.66	5 (45.5)	3 (27.3)	3 (27.3)
P6.	0.00 (1.00)	1.00	4 (36.4)	5 (45.5)	2 (18.2)
P7.	0.09 (0.83)	0.85	3 (27.3)	7 (63.6)	1 (9.1)
P8.	0.27 (0.91)	0.37	5 (45.5)	5 (45.5)	1 (9.1)
P9.	0.64 (0.67)	0.03	6 (54.5)	5 (45.5)	0 (0.0)
MCPIS-9					
M1.	0.00 (0.63)	1.00	2 (18.2)	7 (63.6)	2 (18.2)
M2.	−0.27 (0.65)	0.23	1 (9.1)	6 (54.5)	4 (36.4)
M3.	−1.27 (1.68)^a^	0.05	2 (18.2)	3 (27.3)	6 (54.5)
M4.	−0.82 (1.47)^a^	0.11	2 (18.2)	4 (36.4)	5 (45.5)
M5.	−0.82 (1.25)^a^	0.09	0 (0.0)	7 (63.6)	4 (36.4)
M6.	−0.09 (0.70)	0.77	2 (18.2)	6 (54.5)	3 (27.3)
M7.	−0.09 (0.70)	0.77	2 (18.2)	6 (54.5)	3 (27.3)
M8.	−0.27 (0.47)	0.15	0 (0.0)	8 (72.7)	3 (27.3)
M9.	−0.46 (0.69)	0.09	0 (0.0)	7 (63.6)	4 (36.4)

^a^ Reverse scored; therefore, a negative mean change indicates a decrease in PIF from baseline to endpoint.

For the MCPIS-9 responses, most of the 11 student pharmacists showed no significant change from baseline to endpoint after completing the TLT-based PIF toolkit, with only one question showing significance. The mean change in scores from baseline to endpoint ranged from 0 to 1.27 across all nine components, with a significant difference found in the question related to students feeling ashamed of studying pharmacy (−1.27 [p = 0.049]). No other questions showed significant differences in survey scores. Using the descriptive coding, none of the questions demonstrated a majority of PIF progression among students. Most questions showed PIF stasis, while PIF regression was most common in relation to feelings of shame about studying pharmacy and making excuses for belonging to the profession. See Table 4 for all primary outcome results.

### Secondary outcomes

Among the 39 students who completed both questionnaires only at baseline and midpoint, most of their PSIQ-9 responses showed no change after completing half of the TLT-based toolkit. The mean increase in scores from baseline to midpoint ranged from 0.10 to 0.49 across all nine components, with significant increases found in questions related to communicating with patients (+0.46 [p = 0.011), using patient records (+0.49 [p = 0.036]), and teaching others (+0.44 [p = 0.021]). Descriptively, most students demonstrated PIF stasis across all questions, though more students showed PIF development than PIF regression. Approximately 15–23% of students selected “N/A” as an answer choice at either baseline or midpoint.

Regarding the MCPIS-9 responses, most of the 39 students showed no significant change between baseline and midpoint after completing half of the TLT-based PIF toolkit. The mean change in scores ranged from −0.28 to +0.05 across all nine components, with no significant results. Descriptively, most students exhibited PIF stasis for all questions. For most questions, more students demonstrated PIF regression than PIF progression. However, regarding feelings of belonging to the pharmacy profession, more students showed PIF progression than PIF regression and equal numbers demonstrated PIF progression and regression in terms of identifying positively with members of the pharmacy profession.

Among the 11 student pharmacists who completed both questionnaires at baseline, midpoint, and endpoint, there were no significant results for either questionnaire from midpoint to endpoint after completing the TLT-based PIF toolkit. For the PSIQ-9 questionnaire, the change in mean scores from midpoint to endpoint ranged from −0.73 to +0.09, with no significant results. For the MCPIS-9 questionnaire, the change in mean scores from midpoint to endpoint ranged from −0.73 to +0.27, again with no significant results. See Table 5 for all secondary outcomes in more detail.

**TABLE 5:** Secondary outcomes from baseline to midpoint and midpoint to endpoint.

	Baseline to midpoint (n = 39)						Midpoint to endpoint (n = 11)	
	Mean change (±SE)	*P* value	No. (%) of students development	No. (%) of students stasis	No. (%) of students regression	No. (%) of students N/A	Mean change (±SE)	*P* value
PSIQ-9								
P1.	0.33 (1.01)	0.05	5 (12.8)	22 (56.4)	3 (7.7)	9 (23.1)	0.09 (1.64)	0.67
P2.	0.46 (1.05)	0.01	10 (25.6)	17 (43.6)	3 (7.7)	9 (23.1)	−0.18 (1.83)	1.00
P3.	0.31 (1.10)	0.11	8 (20.5)	18 (46.2)	5 (12.8)	8 (20.5)	−0.46 (1.70)	0.50
P4.	0.46 (1.48)	0.08	11 (28.2)	18 (46.2)	3 (7.7)	7 (18.0)	−0.55 (1.57)	0.29
P5.	0.10 (1.79)	0.78	10 (25.6)	13 (33.3)	7 (18.0)	9 (23.1)	−0.73 (1.79)	0.20
P6.	0.49 (1.37)	0.04	10 (25.6)	18 (46.2)	3 (7.7)	8 (20.5)	−0.64 (1.69)	0.24
P7.	0.26 (0.85)	0.07	5 (12.8)	25 (64.1)	0 (0.0)	9 (23.1)	−0.27 (1.35)	0.59
P8.	0.28 (1.23)	0.11	7 (18.0)	23 (59.0)	2 (5.1)	7 (18.0)	−0.18 (1.60)	0.89
P9.	0.44 (1.21)	0.02	10 (25.6)	21 (53.9)	2 (5.1)	6 (15.4)	−0.27 (1.62)	0.75
MCPIS-9								
M1.	0.05 (0.69)	0.66	8 (20.5)	24 (61.6)	7 (18.0)	N/A	0.00 (0.78)	1.00
M2.	−0.05 (0.65)	0.64	5 (12.8)	26 (66.7)	8 (20.5)		−0.18 (0.41)	0.35
M3.	−0.18 (1.23)^a^	0.25	7 (18.0)	23 (59.0)	9 (23.1)		−0.73 (1.35)^a^	0.13
M4.	−0.23 (1.31)^a^	0.23	6 (15.4)	23 (59.0)	10 (25.6)		−0.55 (2.02)^a^	0.40
M5.	−0.28 (1.32)^a^	0.22	3 (7.7)	26 (66.7)	10 (25.6)		−0.27 (1.90)^a^	0.52
M6.	0.03 (0.74)	0.83	7 (18.0)	24 (61.6)	8 (20.5)		−0.18 (0.41)	0.35
M7.	0.05 (0.83)	0.69	10 (25.6)	19 (48.7)	10 (25.6)		−0.09 (0.70)	0.77
M8.	−0.05 (0.46)	0.53	3 (7.7)	31 (79.5)	5 (12.8)		0.27 (0.47)	0.15
M9.	−0.18 (0.76)	0.18	3 (7.7)	25 (64.1)	11 (28.2)		−0.18 (0.60)	0.42

^a^ Reverse scored; therefore, a negative mean change indicates a decrease in PIF from week 1–8.

## Discussion

Completion of the TLT-based PIF toolkit did not significantly change PI according to the PSIQ-9 and MCPIS-9 questionnaires from baseline to endpoint. A descriptive analysis of the questionnaires showed that the majority of participants demonstrated stasis by the endpoint. However, some questions on the PSIQ-9 questionnaire indicated PIF progression, such as teaching others, while PIF regression was observed on the MCPIS-9 questionnaire, specifically in relation to feelings of shame about studying for the pharmacy profession.

These findings echo prior studies, such as those by Mylrea et al. and Bacci et al., which found minimal measurable change in PIF using the questionnaires [6, 26]. This raises an important methodological concern: if PIF is a subtle, nonlinear, and individualized process, often requiring long-term engagement and context-specific reflection, then the utility of brief, decontextualized quantitative instruments like the PSIQ-9 and MCPIS-9 may be inherently limited in capturing such development. As Matthews et al. and Garza et al. have argued, quantitative PIF instruments must be interpreted cautiously, especially when used to assess pedagogies grounded in transformative learning [23, 24]. In this study, the TLT-based Toolkit fostered deep reflection and internal questioning, yet these meaningful cognitive and emotional shifts may not align with the static, Likert-based structure of the questionnaires.

The finding of PIF regression, particularly around shame toward the pharmacy profession, is especially noteworthy. While it may appear incidental, it likely reflects the core mechanism of Mezirow’s TLT: students encountering disorienting dilemmas that challenge their existing assumptions and provoke discomfort as a precursor to growth [16]. However, due to this being a non-facilitated reflection, some students may have been left with unresolved dissonance or confusion about their roles as future professionals. This underscores a critical design implication: transformative exercises require structured reflection, supportive faculty guidance, and discussion spaces to help students reconstruct meaning from discomfort. Without this support, identity regression or uncertainty may occur.

Taken together, these findings suggest that while the PSIQ-9 and MCPIS-9 offer a starting point for exploring PIF, they may not be suitable standalone tools for evaluating short-term, reflection-based interventions. The subtle shifts in PIF seen through descriptive analysis and narrative responses indicate that qualitative data, currently under analysis, may yield a richer, more authentic understanding of how students engaged with the toolkit and experienced professional growth or dissonance. Future studies should consider using qualitative tools such as reflective narratives, discourse analysis, or semi-structured interviews to better capture the complexity and developmental trajectory of PIF in pharmacy students.

### Limitations

There were several limitations in this study. The first limitation is that only 14% (11 out of 79) of eligible students participated across all time points. This is likely because completing the questionnaires was not a requirement for course completion and students may not have perceived a benefit. As a result, the sample size is small and may not be generalizable to the entire class or other P2 students.

Additionally, our study, similar to others [21, 27], experienced lower response rates as the study progressed, which may be attributed to questionnaire fatigue, especially at the endpoint, since students were simultaneously completing multiple end-of-semester course and instructor evaluations. Despite providing 10 min of class time to complete both questionnaires, it remains unclear how students utilized this time. Some questionnaires were also incomplete and thus not included in the results, further contributing to the small number of students in this study.

Another limitation is that the original validated PSIQ-9 questionnaire included “N/A” as an answer choice. As a result, in our secondary outcomes, 15%–23% of students selected “N/A”; had this option not been available, we might have gained a better understanding of their PI, as they would have been required to choose from the other available options. Additionally, the phrasing of questions in the MCPIS-9 questionnaire may have confused students if they did not read them carefully, as questions 3–5 were negatively worded. For example, if a student quickly selected “5” (strongly agree) for all the questions without reading carefully, they would indicate that they “strongly agree” with being ashamed of studying for the pharmacy profession in question 3. If this happened with multiple students, our primary outcome showing PIF regression in questions 3 and 4 of the MCPIS-9 questionnaire might be inaccurate.

Moreover, this study could not account for potential confounders. Many factors outside of the TLT-based PIF toolkit might influence students’ PI. For example, positive and negative experiences during their once-weekly IPPE rotations, other coursework, and even the news may impact PI. Research by Dee et al. found that more than 51% of pharmacists experience burnout [4], which is also highlighted in the news, such as reports of nationwide walkouts by community pharmacists at large corporations [28]. Therefore, students’ scores on the questionnaires might have remained the same or regressed from baseline to endpoint due to negative experiences outside of the TLT-based PIF toolkit.

### Future considerations

The lack of significant change in PIF, as measured by the PSIQ-9 and MCPIS-9, coupled with the controversial and declining use of these quantitative instruments in recent literature [23, 24], suggests that these tools may not be appropriate for evaluating short-term, reflection-based interventions in pharmacy students. While both instruments have demonstrated past utility in broader health professional education [21, 22, 27], their ability to detect the nuanced, individualized, and developmental shifts inherent to PIF is limited. The findings from this study support the growing argument that these quantitative tools may lack the sensitivity needed to capture the depth of change prompted through reflection [23, 24], and future use of the PSIQ-9 and MCPIS-9 in similar pharmacy education contexts is not recommended.

Rather than focusing solely on quantifiable change, future research should prioritize qualitative methods that align more closely with the core tenets of PIF and TLT. Reflective writing, narrative analysis, and discourse-based interviews offer the opportunity to capture thick descriptions of how students process, internalize, and make meaning of disorienting professional situations. Such approaches are better suited to evaluating the inner transformation students may experience, especially in cases where discomfort, value conflicts, or uncertainty arise. The rich data currently being analyzed from students’ reflective responses to the TLT-based toolkit is expected to offer greater insight into these developmental processes.

In addition, future iterations of the toolkit should incorporate structured debriefing led by pharmacist facilitators. While the toolkit prompted internal reflection, the absence of guided discussion may have left students with unresolved dissonance or uncertainty about their professional roles. When instructors share their own experiences and model vulnerability, they demonstrate that identity development is an ongoing process [29]. This type of authentic engagement helps normalize ambiguity, fosters psychological safety, and supports students in meaningfully reconstructing their evolving identity as pharmacists.

## Conclusion

There were no significant changes in students’ self-evaluation of PI, as measured by the PSIQ-9 and MCPIS-9 questionnaires, over the 15-week course using the TLT-based toolkit. These findings suggest that such quantitative tools may lack the sensitivity needed to detect the nuanced, individualized nature of PIF. Future studies should consider longitudinal, mixed-methods designs that prioritize qualitative reflection and facilitated dialogue to better capture the transformative potential of disorienting dilemmas within pharmacy education.

## Data Availability

The raw data supporting the conclusions of this article will be made available by the authors, without undue reservation.

## Abbreviations

PIF, Professional identity formation; TLT, Transformative Learning Theory; PSIQ-9, Professional Self Identity Questionnaire; MCPIS-9, Macleod Clark Professional Identity Scale.

## Appendix A: 10 phases of Mezirow’s TLT in the process of simulation-based learning in healthcare education [30]

• Phase 1: defined as a “disorienting dilemma”
• Phase 2: “A self-examination with feelings of guilt or shame”
• Phase 3: “A critical assessment of epistemic, sociocultural, or psychic assumptions”
• Phase 4: “Recognition that one’s discontent and the process of transformation are shared and that others have negotiated a similar change”
• Phase 5: “Exploration of options for new roles, relationships, and actions”
• Phase 6: “Planning of a course of action”
• Phase 7: “Acquisition of knowledge and skills for implementing one’s plans”
• Phase 8: “Provisional trying of new roles”
• Phase 9: “Building of competence and self-confidence in new roles and relationships”
• Phase 10: “A reintegration into one’s life on the basis of conditions dictated by one’s perspective”

Utilizing Transformative Learning Theory to Enhance Professional Identity Formation ^©^ 2025 by Kathryn Sawyer is licensed under CC BY-NC-ND 4.0. To view a copy of this license, visit https://creativecommons.org/licenses/by-nc-nd/4.0/.
